# Demand-oriented design of telemedical services in gynecologic oncology

**DOI:** 10.1186/s12913-023-10176-5

**Published:** 2023-10-27

**Authors:** Sascha Hoffmann, Julia Beissner, Rebekka Hieber, Johann Jacoby, Tobias Engler, Christina Barbara Walter

**Affiliations:** 1 Department of Women’s Health, University Women’s Hospital Tübingen, Calwerstr. 7, 72076 Tübingen, Germany; 2 Institute for Clinical Epidemiology and Applied Biometry, University of Tübingen, Silcherstr. 5, 72076 Tübingen, Germany

**Keywords:** Telemedicine, Gynecology, Oncology, Patient compliance, Supportive care

## Abstract

**Background:**

The medical field is in the midst of a massive expansion in telemedical services. However, it is not possible to say to what extent telemedical offerings can be designed to meet needs in the German healthcare system. This study provides insights into demand-oriented care using telemedical services for gynecological patients.

**Methods:**

A total of 262 patients who received systemic therapy for gynecological oncology were surveyed anonymously using a questionnaire regarding their acceptance of telemedicine from February 2021 to April 2021.

**Results:**

Insufficient computer skills were associated with less acceptance of telemedicine treatment by gynecological oncology patients and presented a barrier. However, the patient’s level of education was not related to the level of acceptance. Long travel distances from medical facilities and some types of patient occupations significantly increased the acceptance of telemedicine services. A high level of education, on the other hand, was not associated with the approval of telemedical approaches. Long journeys and work commitments increased the acceptance of telemedical visits.

**Conclusions:**

The results of this study show that the factors investigated have an influence on the acceptance of telemedical offerings by patients. Barriers such as insufficient computer skills must be taken into account when implementing telemedicine services. Telemedicine can provide physical and economic relief for patients if telemedical planning is tailored to their needs.

## Introduction

Telemedicine is an open and constantly evolving science as it incorporates new advancements in technology and responds to the changing health needs and contexts of society. Four elements are relevant to telemedicine: its purpose is to provide clinical support; it aims to overcome geographical barriers by connecting users and providers who are not in the same physical location; it involves the use of different types of information and communication technologies; its aim is to improve health outcomes [1]. In general, telemedicine has changed structural and process aspects of primary care, such as the physical space in which patients are treated, and created a new environment to which both patients and providers must adapt, so it is important to recognize the opportunities and limitations of this new technology to help providers maintain the kind of personalized care that patients expect and that helps build relationships [2].

The importance of telemedicine approaches has gained additional significance, particularly as a result of the 2019 COVID-19 pandemic [3–5]. Overall, provider use and perceptions related to using virtual care have improved over time, and providers adapted quickly to providing virtual care during COVID-19 and made plans to provide virtual care long-term [6]. And from a patient perspective, it has been shown that despite the problems with or drawbacks of delivering tele-complementary and integrative health (CIH) therapies, veterans’ use of CIH therapies increased substantially during the COVID-19 pandemic [7]. In addition, it has been shown that the integration of general digital technologies into pandemic policy and control may have been one of several distinguishing features of countries that flattened their COVID-19 incidence curves and kept mortality rates low [8]. These findings, which are very much due to the circumstances of the COVID-19 pandemic, give a sense of the potential that lies in digital offerings and, in particular, telemedicine approaches.

Telemedicine can be used across all medical disciplines. A 2020 review determined that the COVID-19 pandemic has changed the delivery of medical services, with applications for telemedicine in different areas of medical practice, distinguishing four sectors: out-patient consultation; inpatient care (emergency triage, hospitalization, interhospital consultation); patient and physician satisfaction; education [9]. Some specialties have gained particularly great benefits from the implementation of telemedical services, specifically primary care, mental health, dermatology, radiology, chronic disease management, pediatrics, genetics, and genetic counseling.

Due to the varying requirements in the different treatment phases of oncological diseases, there is a need for differentiated telemedical strategies to support patients and families during the entire oncological course of the disease [10]. For example, it has been shown that breast cancer patients consider telemedicine to be beneficial and that patient satisfaction correlates with the user-friendliness of telemedicine [11]. A paper from Qatar reports that eConsult services offer the potential to improve access, interdisciplinary communication, and patient and provider satisfaction [12], and a paper from Canada indicates that digital consultations reduce waiting times for conventional consultations [13]. A scoping review showed that both primary care physicians and patients can be satisfied with video consultations in general practice in certain contexts of use and that appropriate clinical decision-making is possible with them, but drawbacks such as a deteriorating relationship between physician and patient were also highlighted [14]. Several scoping reviews have shown that for gynecological oncology, including breast cancer care, teleconsultation is used in particular during treatments and follow-up; despite the acceptance of telemedicine in principle, a skeptical attitude from the patient side was also described in individual studies [15–17].

These results show that telemedicine can be used beneficially. This study intends to provide findings for needs-based care through telemedicine services for gynecological oncology patients receiving systemic therapy.

## Methods

For data collection, 262 patients undergoing adjuvant or palliative drug treatment for breast cancer or a gynecological malignancy at an oncological day clinic were interviewed between February 2021 and April 2021. The questionnaire used was self-created. The questionnaire was completely anonymous and voluntary.

A vote of the ethics committee of the University of Tübingen (902/2020BO2) was obtained to conduct the survey.

Data management was done with REDCap (V 9.8.5) and Microsoft Excel (Office 2019), and static analysis was performed using JMP (V 15.2.0) to generate frequency and contingency tables and to calculate the test for dependence in **χ**2-tests. The significance threshold was set at α = 0.05.

### Description of the collective

The adjuvant or palliative treatment of patients in an oncological day clinic was due to breast carcinoma (n = 206; 78.6%), ovarian carcinoma (n = 28; 10.7%), cervical carcinoma (n = 5; 1.9%), endometrial carcinoma (n = 8; 3.1%), or an unspecified reason (n = 15; 5.7%).

**Fig. 1:**
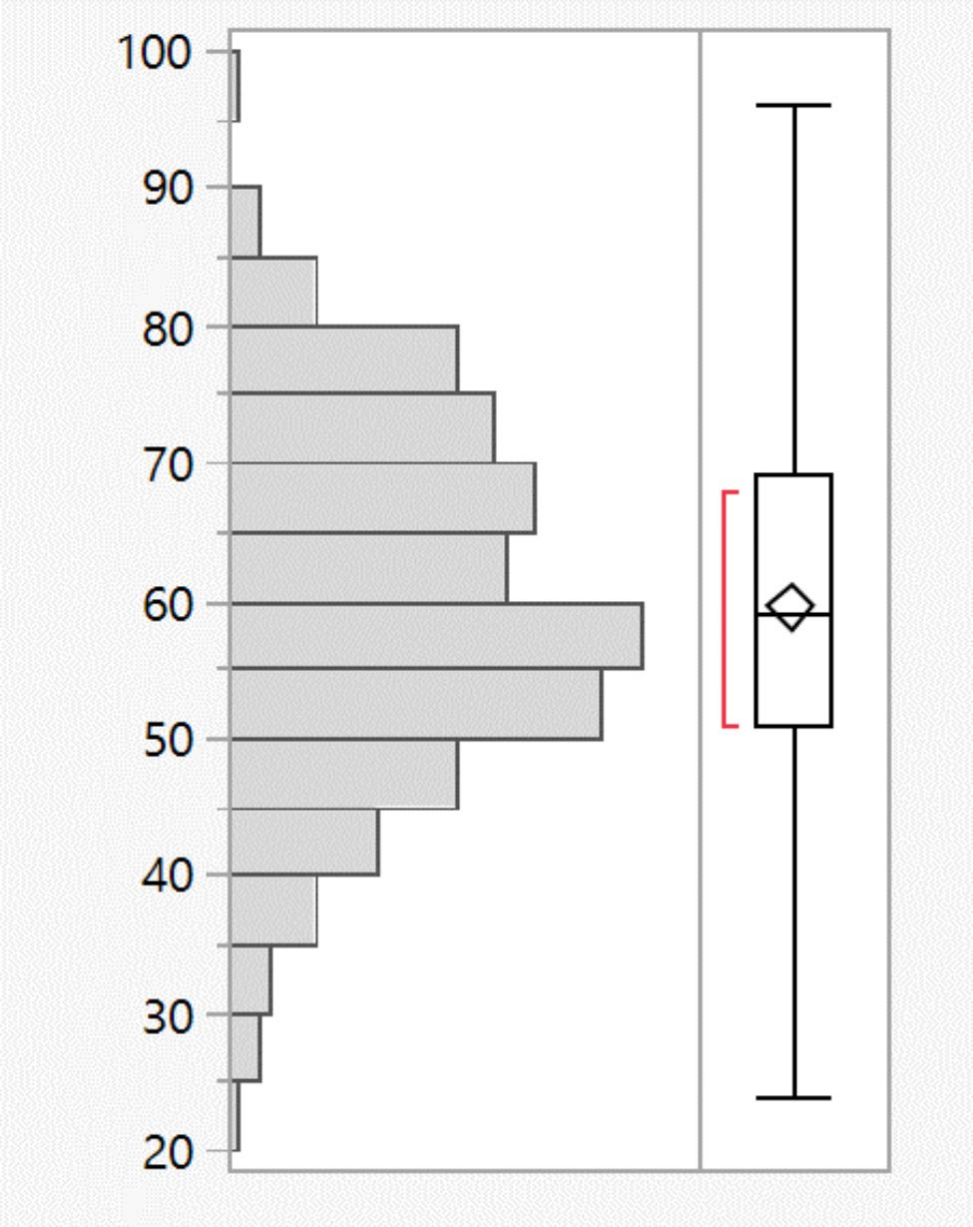
Distribution of age in years of life

The youngest patient was 24 years old, and the oldest was 96 years old. The average patient age was 59.5 years (M = 59.5; SD = 12.9) (Fig. 1).

## Results

The current subjective health assessment was collected using the EQ VAS (visual analog scale). The respondents marked their health status on a scale from 0 to 100. The mean value of the EQ VAS was 58.5 (SD = 22.0; 95% CI = 55.8–61.3).

Of the patients surveyed, n = 144 (55.0%) stated that they were “very satisfied” with the treatment so far, n = 112 (42.7%) answered “strongly agree” to the question of whether they were satisfied with the treatment, and n = 1 (0.4%) answered “strongly disagree.” No patient chose the option of being “not at all” satisfied, and n = 5 respondents (2.0%) did not respond. Respondents who were not satisfied with the treatment could explain their dissatisfaction by typing in a comment. Thirty-two patients answered this question, some of them with multiple responses. The table shows the list of responses (Table 1).

**Table 1:** Reasons for dissatisfaction

Reasons for dissatisfaction	number of mentions
Waiting times	n = 11
Too little information or education	n = 6
Poor accessibility	n = 5
Difficult communication	n = 4
Change in contact persons	n = 3
Little time for conversation	n = 2
Scheduling of appointments	n = 3
No telephone conversations offered	n = 1
Therapy did not work	n = 1
Referred to doctors in private practice, despite having their own specialist department	n = 1
Location for treatment	n = 1
Findings not handed out	n = 1
Lack of staff	n = 1
Discharged too soon after an operation	n = 1

Of the respondents (n = 262), n = 149 (56.9%) stated that they had personally informed themselves on the internet about their disease, n = 127 (48.5%) about their treatment options, and n = 59 (22.6%) about different treating hospitals.

**Fig. 2:**
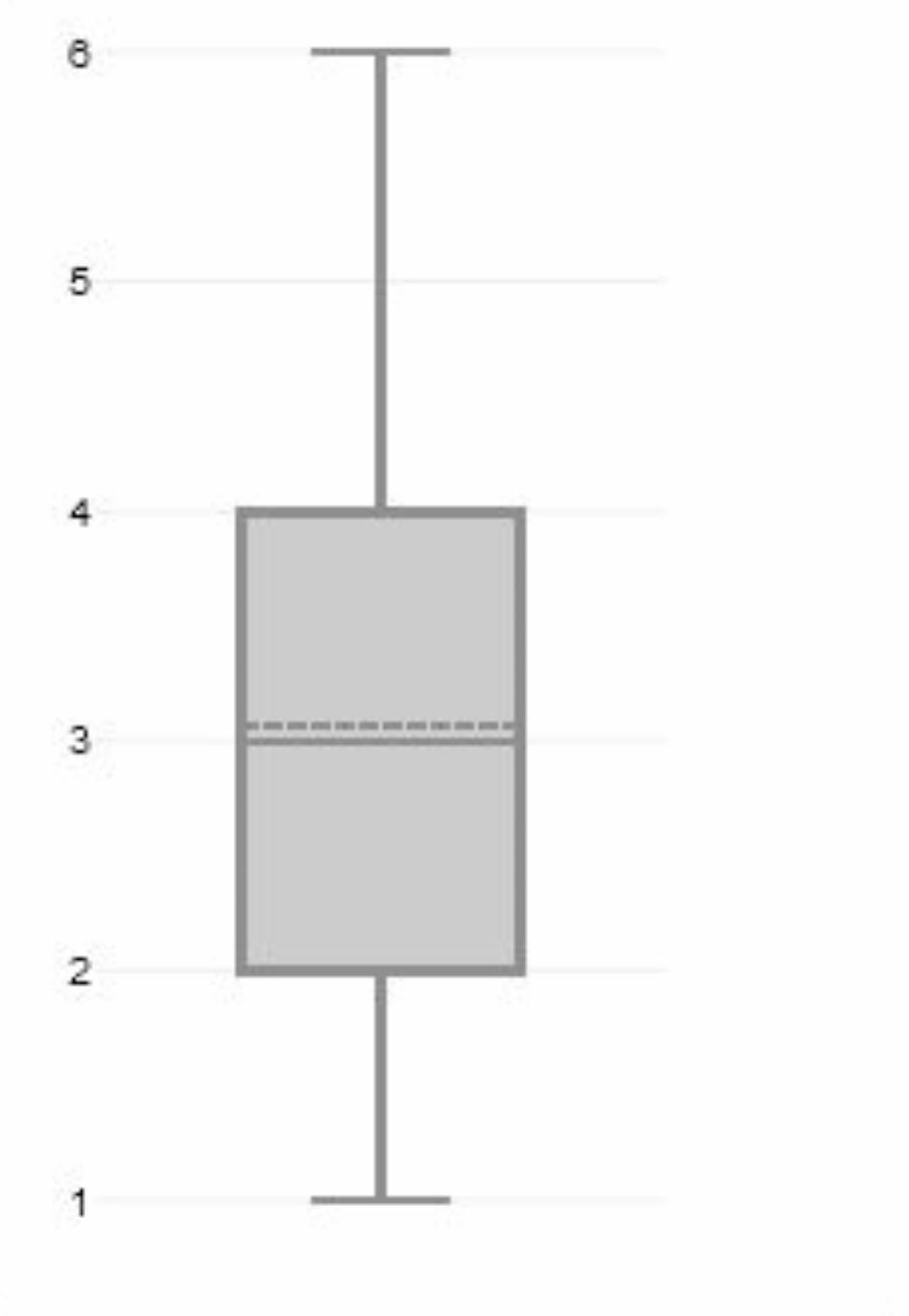
Assessment of the improvement of medical care for female cancer patients in Germany because of telemedical offerings (M = 3.06; Md = 3). Indication is in whole number increments from 1 = strongly agree to 6 = completely disagree

Respondents were asked to indicate, on a scale of 1 to 6, whether they felt that telemedicine services improved medical care for female cancer patients, with 1 indicating “completely agree” and 6 “completely disagree” (Fig. 2).

The following were mentioned as possible specific forms of telemedical communication:

- Telephone.
- Video call provider (e.g., Skype).
- Email.
- App (smartphone/tablet).
- SMS (short message service).
- Other.

**Fig. 3:**
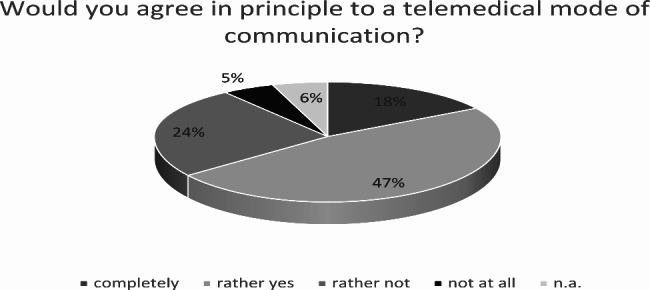
Basic approval of telemedical forms of communication

Such forms of communication were generally agreed to, with n = 46 (17.6%) respondents replying “completely” and n = 124 (47.3%) replying “yes,” whereas n = 14 (5.3%) agreed “not at all” and n = 62 (23.7%) replied “rather not.” No answer was given by n = 16 (6.1%) of the respondents (Fig. 3).

The average distance traveled for (n = 249; not specified for n = 13) was 33.6 km (M = 33.6; SD = 27.5; 95% CI = 30.1–37.0). N = 214 (81.7%) of the respondents stated that the journey was “not at all stressful” or “not stressful” for them, n = 41 (15.6%) described the journey as “very stressful” (n = 7; 2.7%) or “somewhat stressful” (n = 34; 13.0%), and no information was given by n = 7 (2.7%) respondents. For the group “not at all or not stressful,” the data of n = 205 (78.2%) of the respondents resulted in an average travel distance of M = 29.7 km (SD = 22.8; 95% CI = 26.5–32.8). In contrast, the average travel distance in the group “a very stressful or somewhat stressful travel distance” (n = 40; 15.3%) was 54.2 km (M = 54.2; SD = 39.1; 95% CI = 41.7–66.7). An *χ*2-test was performed to test whether there was a relationship between a travel burden and consent for telemedicine treatments. No expected cell frequencies were less than 5. A statistically significant association was found (p = .013).

Of the respondents, n = 29 (11.1%) stated that they had beginner computer skills, n = 79 (30.2%) had amateur skills, n = 105 (40.1%) had advanced skills, and n = 12 (4.6%) had professional skills. No response was given by n = 37 (14.1%). An χ2-test was conducted to test whether there was an association between computer literacy skills and consent for telemedicine treatments. No expected cell frequencies were less than 5. A statistically significant relationship was found (p = .001).

N = 137 (52.3%) stated that they had completed vocational training, n = 83 (31.7%) that they had completed a degree, n = 20 (7.6%) that they had not completed any further education, n = 11 (4.2%) that they had completed a doctorate or habilitation, and n = 3 (1.1%) that they had completed another degree. No answer was given by n = 8 (3.1%) of the respondents. An χ 2-test was conducted to test whether there was a relationship between type of educational qualification and consent for telemedicine treatments. No expected cell frequencies were less than 5. No statistically significant association was found (p = .1744).

Regarding employment status, the majority of patients reported being “retired” (n = 109; 41.6%), no answer was given by n = 69 respondents (26.3%), n = 53 (20.2%) selected “other,” n = 21 (8.0%) chose “housewife,” n = 8 (3.1%) indicated they were “unemployed,” and n = 2 (0.8%) selected “student.” At the time of the survey, n = 50 (19.2%) of respondents were employed full time, n = 64 (24.5%) were employed part time, n = 130 (49.8%) were not employed, and n = 17 (6.5%) did not specify. An χ 2-test was conducted to test whether there was an association between employment and consent for telemedicine consultations. No expected cell frequencies were less than 5. No statistically significant association was found (p = .004).

Table 2 displays the relationship between approval of telemedical communication and commute to treatment facility, computer skills, and employment, line-by-line with percentages in parentheses.

**Table 2:** Relationship between approval of telemedical communication and commute, computer skills, and employment, line-by-line with percentages in parentheses

	Would you agree in principle to a telemedical mode of communication?				
		Yes	Rather not	Total	
**When you think about the journey from your home to the clinic: How strenuous/stressful do you find the journey?**	Stressful	31 (86.1%)	5 (13.9%)	36	
	Not stressful	134 (65.4%)	71 (34.6%)	205	*p = .013*
**Computer skills**	Beginner or lay knowledge	60 (57.1%)	45 (42.9%)	105	
	Advanced or professional knowledge	94 (81.7%)	21 (18.3%)	115	*p = .001*
**Employment**	Part-time or full-time employment	24 (10.3%)	87 (37.2%)	111	
	Not employed	48 (20.5%)	75 (32.0%)	123	*p = .004*

## Discussion

Telemedicine in oncology enables direct interaction with the patient in real time and video conferencing with transmission of laboratory, imaging, and pathology data. In oncology in general, the remote monitoring of therapy side effects [18, 19], psychological support [20], palliative care provision [21], management of patients’ symptoms [22], and enrollment and follow-up assessment in clinical trials [23] are known as applications.

Telemedicine applications are already widely used in gynecology. Specifically, the use of telemedicine in gynecology is beneficial in screening, prevention, family planning, mental health, prescriptions, and procedures [24]. Telemedical applications for telemedicine in gynecologic cancer care exist for the prediagnosis, pretreatment, treatment, and post-treatment/survivorship phases of cancer care [25].

A particular increase in telemedicine offerings was observed as a result of the COVID-19 pandemic, with evidence of good user adoption [26–28]. Even when very strong tendencies for good patient satisfaction could be found, these publications also require analysis regarding the different factors involved in the acceptance of telemedical offerings. Also, our work shows a generally positive evaluation of telemedical consultations by the patients and shows, furthermore, that different factors influence the acceptance, sometimes significantly. In principle, telemedicine seems to be effective for oncological patients from the point of view of those treating them [29]. Telemedicine has proven to be beneficial, cost-effective, and satisfactory to patients and providers for various medical conditions unless a physical examination is needed [30].

Dholakia et al. described a high level of approval for telemedicine among gynecologic patients, while Nestlerode et al. described a negative attitude among patients [16, 31]. This contradiction may have been caused by factors that constitute a need-based service. Therefore, the results of this study may help optimize the effectiveness of telemedicine using a targeted application.

The results of the present study showed that the gynecological oncology patients surveyed (n = 256; 97.7%) were already highly satisfied with their treatment without the use of systematic telemedicine approaches. The majority of the respondents, nevertheless, stated that they agreed with telemedical communication (n = 170; 64.9%), whereas n = 76 (29.1%) respondents stated that they did not agree with it. Thus, the challenges for telemedicine approaches arise from the fact that conventional treatment already sets a high standard and, on the other hand, that some patients are not open to telemedicine approaches.

Bizot et al. noted that face-to-face visits are still the standard for breast cancer patients but also showed that there are population groups for whom an equivalent telemedicine approach could be considered [32]. According to their study, further steps should describe the general mix of different consultation modalities and compare the satisfaction scores of face-to-face visits and teleconsultations via telephone or video call [32].

One study found that telemedicine can reduce days missed from work or school [33]. Complementing this, our results show a significantly increased acceptance of telemedicine among working patients. In our view, this seems to be justified by the economic benefits and may therefore even be positive for the healthcare system as a whole. Patients using teleconsultations attended fewer appointments, with significant cost-savings per person driven mainly by reduced travel and parking costs [34]. Satisfaction with telemedicine services is high when patients have shorter travel times and lower costs [35, 36].

This can be confirmed by the present study, as a significant correlation was found between the length of the journey to the clinic and approval of telemedicine services (p = .013); avoiding a long treatment journey can therefore relieve patients somewhat, save transport costs, and conserve natural resources.

The present study also shows stronger approval for telemedical treatment options from the group of respondents with advanced or professional computer skills than respondents with beginner or lay skills. This has also been confirmed by different studies internationally [37, 38].

Scott-Kruse et al. identified education level as a possible barrier to telemedicine services [39]. However, this study was unable to establish a statistical correlation between educational attainment and approval of telemedicine services (p = .1744). This is attributed to the fact that educational attainment cannot be equated with computer skills, and a differentiated consideration is necessary in this regard.

For gynecological oncology patients, telemedicine was shown to be a useful platform for cancer care across the spectrum of social vulnerability during the pandemic and beyond [40]. Employment is also affected by oncological therapy; interestingly, employed patients showed significantly higher approval for telemedicine approaches (p = .004). The economic security of patients could be strengthened by facilitating the reconciliation of employment and medical treatment, which can be achieved by simplifying the availability of the treatment.

A systematic review created a heterogeneous picture regarding the acceptability of and satisfaction with telemedicine in 2022 [41]. Our study may offer an explanation for this: a needs-based design is likely to be a determining factor for acceptance and satisfaction.

Overall, the results show different patients have different demands for telemedical services, which should be identified to ensure needs-based telemedical care. In this context, Traulsen et al. described a proposal in which quality criteria could be collected through continuous evaluation under the aspect of good and safe patient care [42].

### Limitations of this study

A limitation of this study is that, due to the limited sample size, stratification of patients by different clinical situations was not possible. It would be interesting for future studies to analyze different cancer types and different treatment approaches in balanced samples because we think that it is very likely that the type of disease also has influences on the requirements and acceptance of telemedicine. In addition, it should be noted that this is an all-female collective from a unicenter study. In order to understand this in more detail, a larger sample size in a multicenter follow-up study would be helpful.

## Conclusions

A high level of education is not associated with a higher approval of telemedicine approaches. Having insufficient computer skills, however, increases the rejection of telemedical treatment by gynecological oncology patients. It can be concluded that before offering telemedicine treatment, it should be verified that patients are technically competent. In addition, acceptance of telemedicine services is higher among patients who have a long commute and who are employed. Targeted offers for these patient groups can prove to be both ecologically and economically beneficial, as the burden on the environment can be reduced, and patients are economically better off by retaining their employment during therapy. This in turn reduces the burden on the environment and the healthcare system.

Further studies should be carried out to develop a demand-oriented design of telemedical services.

## Data Availability

The data sets generated and/or analysed in this study are available from the corresponding author sascha.hoffmann@med.uni-tuebingen.de.
